# High-precision neural stimulation through optoacoustic emitters

**DOI:** 10.1117/1.NPh.9.3.032207

**Published:** 2022-03-23

**Authors:** Linli Shi, Ying Jiang, Nan Zheng, Ji-Xin Cheng, Chen Yang

**Affiliations:** a Boston University, Department of Chemistry, Boston, Massachusetts, United States; b Boston University, Department of Biomedical Engineering, Boston, Massachusetts, United States; c Boston University, Division of Materials Science and Engineering, Boston, Massachusetts, United States; d Boston University, Department of Electrical and Computer Engineering, Boston, Massachusetts, United States

**Keywords:** optoacoustic, neural stimulation, ultrasound, fiber, nanoparticles

## Abstract

Neuromodulation poses an invaluable role in deciphering neural circuits and exploring clinical treatment of neurological diseases. Optoacoustic neuromodulation is an emerging modality benefiting from the merits of ultrasound with high penetration depth as well as the merits of photons with high spatial precision. We summarize recent development in a variety of optoacoustic platforms for neural modulation, including fiber, film, and nanotransducer-based devices, highlighting the key advantages of each platform. The possible mechanisms and main barriers for optoacoustics as a viable neuromodulation tool are discussed. Future directions in fundamental and translational research are proposed.

## 1 Introduction

Neuromodulation at high spatial precision poses great significance in understanding the flow of information in the nervous system, thus providing enticing opportunities to decipher and manipulate the intricate organization of the mammalian brain. Clinically, neural stimulation has been successfully applied for treating neurological and psychiatric disorders. For example, electrode-based deep brain stimulation (DBS) has been applied to humans with epilepsy,^1^ Alzheimer’s disease,^2^ Parkinson’s disease,^3^ and treatment-resistant depression.^4^ Transcranial magnetic stimulation has been used for major depression^5^ and transcranial direct current stimulation works for the treatment of anxiety,^6^ pain,^7^ and chronic motor stroke.^7^ Besides, vision restoration has been achieved by stimulation of retinal ganglion cells (RGC) evoked by the optogenetic actuator ChrimsonR (or ChrimsonR fused to tdTomato, ChR-tdT), which served as an appealing modality in non-human primates^8^^,^^9^ and blind patients^10^ with ongoing clinical trials (ClinicalTrials.gov identifier NCT02556736). Toward surgical applications, electrical stimulation of the individual dorsal root nerves has been applied to identify the abnormal one in selective dorsal rhizotomy (SDR) on children.^11^^,^^12^

Electrical stimulation, as the most prescribed neuromodulation method clinically, has been used for treating neurological disorders.^13^^–^^15^ It is limited by the invasive nature of electrode implantation^16^ and poor spatial resolution due to the current spread.^17^ As a rapidly growing modality, optogenetics has been harnessed in a myriad of brain neuromodulation studies in rodents with high precision and cell-type specificity.^18^^,^^19^ However, the requirement of viral transfection hinders its broad application in humans.^20^ Toward nongenetic stimulation, photothermal neural stimulation^21^^–^^23^ has attracted increasing interest in basic science and translational applications,^24^^,^^25^ where the associated thermal toxicity raises a concern of tissue damage.^26^ Focused ultrasound neuromodulation,^27^^–^^29^ another emerging modulation modality, has been applied in rodents,^28^^,^^30^ rabbits,^31^ non-human primates,^32^ and humans,^27^ given its noninvasive nature with a deep penetration depth.^33^ Nevertheless, the lateral spatial resolution of focused ultrasound suffers from the acoustic wave diffraction limit at the level of several millimeters.^27^ As the ultrasound neuromodulation rapidly advances for fundamental and clinical studies, the limited spatial precision remains as a challenge, hindering it from applications requiring single neuron/nerve stimulation, such as retinal prostheses^34^^,^^35^ and SDR.^11^ New technologies are still sought to achieve genetic-free and precise neural stimulation.

**Fig. 1:**
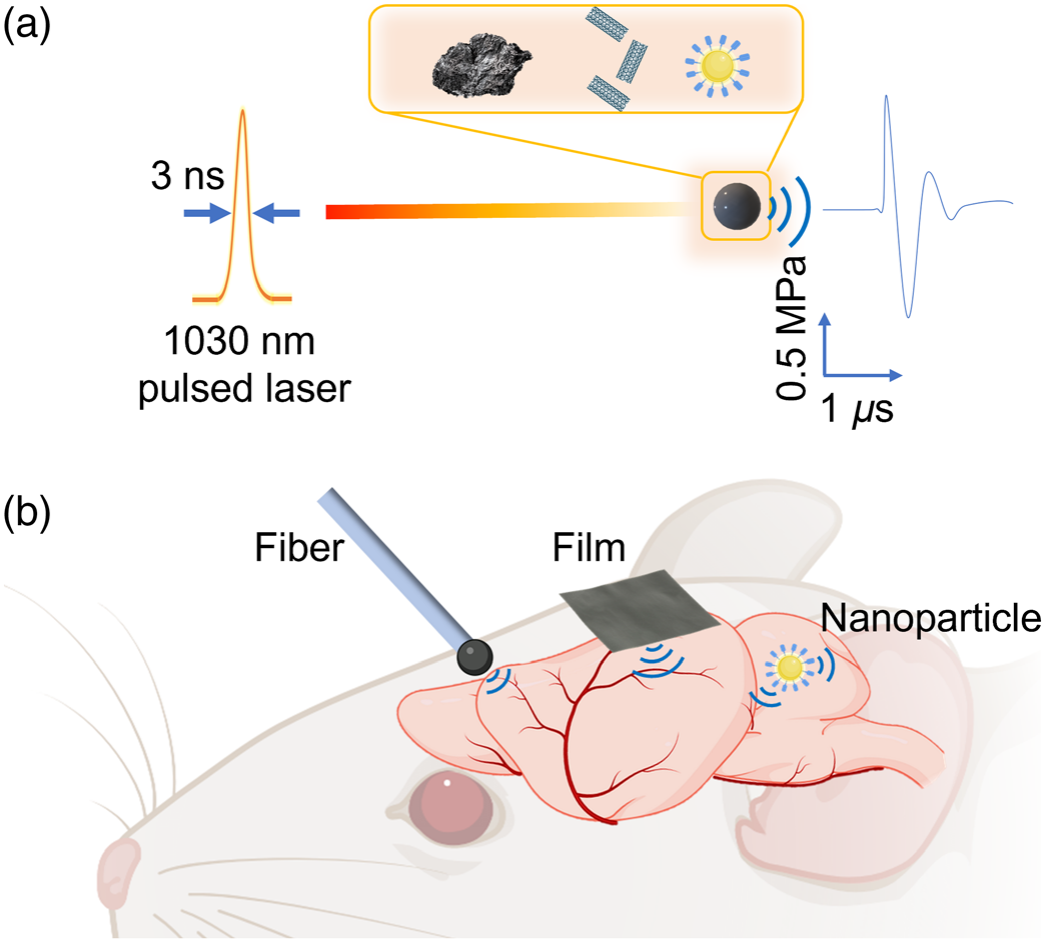
Schematic of optoacoustic neural stimulation. (a) Principle of optoacoustic generation based on varied absorption agents. Inset: Example absorption agents including graphite,^36^ CNT,^37^^,^^38^ and PANs.^39^ (b) Optoacoustic stimulation platforms based on optical fibers, biocompatible films, and nanotransducers. (Created with Ref. 40.)

Alternative to piezo transducers, the optoacoustic technique is a way to generate ultrasound benefiting from the merits of ultrasound with high penetration depth as well as the photons with high spatial precision. The optoacoustic process, in which a pulsed light is illuminated on an absorber, results in transient heating and subsequent generation of acoustic waves at ultrasonic frequencies [Fig. 1(a)]. The life sciences have witnessed rapid development of optoacoustic technologies,^41^ for imaging of living biological structures ranging from subcellular structures to organs^42^ and even whole animals.^42^ Noimark et al.^43^ designed a fiber-based optoacoustic emitter (FOE) and Fabry–Pérot cavity for ultrasound transmission and reception, respectively. It achieved all-optical ultrasound imaging of an aorta. Lan et al. developed an FOE with three ultrasound sensors. By integrating with an augmented reality (AR), this device served as a fast and accurate surgical guidance for tumor removal.^44^ Beyond imaging, in sonoporation, FOE has also been used to transiently increase the cell membrane permeability, which allowed for localized delivery of membrane-impermeable molecules into cells.^45^ Silva et al. used an FOE to promote transfection of DNA encoding GFP into monkey fibroblast cells.^46^ Lee et al. designed an optoacoustic film coated on a concave lens for high-precision focusing, which served as an invisible sonic scalpel to cut pig eyeballs.^47^

Our team recently started to exploit the optoacoustic effect for high-precision neuromodulation. By repurposing the FOE originally developed for surgical guidance, we showed the first example of optoacoustic neurostimulation^36^ [Fig. 1(b), left]. In this work, a light absorber was coated on the fiber tip for localized neuron stimulation at unprecedented submillimeter spatial resolution both *in vitro* and *in vivo*. Since then, our team has further improved the optoacoustic conversion efficiency through rational material design.^45^ More recently, we developed a tapered fiber optoacoustic emitter (TFOE), which pushed the modulation spatial precision to single neuron and subcellular level.^37^ Toward scalable and potentially noninvasive stimulation, a film-based optoacoustic silk scaffold for neural stimulation and regeneration has been developed by Zheng et al.^38^ [Fig. 1(b), middle]. Furthermore, to address the urgent need for noninvasive neuromodulation, semiconducting polymer nanoparticles-based photoacoustic nanotransducers (PANs) were developed by Jiang et al.^39^ [Fig. 1(b), right]. In this paper, we discuss important considerations in device design, potential mechanisms, and the main barriers at present to the uptake of optoacoustics as a viable neuromodulation tool. We also provide an outlook on future directions in fundamental and translational research.

## 2 Working Principle and Key Criteria of Optoacoustic Generation

The general optoacoustic equation for wave generation and propagation in an inviscid medium has been described by Wang and Wu^48^ as

$$(\nabla^{2}-\frac{1}{v_{s}^{2}}\frac{\partial^{2}}{\partial t^{2}})p(\overset{\to }{r},t)=-\frac{\beta }{kv_{s}^{2}}\frac{\partial^{2}T(\overset{\to }{r},t)}{\partial t^{2}}$$ (1)

where $p(\vec{r},t)$ is the acoustic pressure at location r and time t and T is the temperature rise, $v_{s}$ is the speed of sound in medium, β is the thermal expansion coefficient, and k is the isothermal compressibility, which can be expressed as

$$k=\frac{C_{P}}{\rho v_{s}^{2}C_{V}}.$$ (2)

Here, ρ is the mass density, $C_{P}$ and $C_{V}$ are the specific heat capacities at constant pressure and volume, respectively.

The left part of Eq. (1) describes the wave propagation, whereas the right-hand side represents the source term.

As the key criteria for optoacoustic generation, two conditions, namely thermal confinement and stress confinement, must be met.^49^ Toward these criteria, there are two important timescales: thermal and stress relaxation.

The thermal relaxation time, which characterizes the thermal diffusion, is given as

$$\tau_{th}=\frac{d_{c}^{2}}{\alpha_{th}},$$ (3)

where $\alpha_{th}$ is the thermal diffusivity ($m^{2}/s$) and $d_{c}$ is the characteristic dimension of the heated region.

While the stress relaxation time, which characterizes the pressure propagation, is estimated as

$$\tau_{s}=\frac{d_{c}}{v_{s}}.$$ (4)

The thermal confinement criterion is met when the laser pulse duration is much shorter than $\tau_{th}$, and heat conduction is negligible during the laser excitation. Similarly, the stress confinement criterion is met if the laser pulse duration is much shorter than $\tau_{s}$, and stress propagation is negligible during the laser excitation.

Regarding the stress confinement, a laser pulse duration of picosecond or nanosecond is required to build up the thermoelastic pressure. Since a mode-locked picosecond pulsed laser suffers from low pulse energy, Q-switched nanosecond pulsed lasers with a high pulse energy have become popular in optoacoustic applications.^50^ Per the thermal confinement, the laser pulse width needs to be shorter than the thermal conduction time aiming at sufficient efficiency. For example, in the study of a nanoparticle-based scenario,^39^ the thermal diffusion time constant is ∼6  ns, which guided the researchers to use a 3-ns pulsed laser for efficient optoacoustic generation.

On laser excitation, the fractional volume expansion $\frac{dV}{V}$ can be expressed as

$$\frac{dV}{V}=-kp+\beta T.$$ (5)

Upon meeting the condition for both thermal and stress confinements, the fractional volume expansion is negligible. Thus, the local pressure rises $p_{0}$ immediately after the laser pulse can be derived from Eq. (5):

$$p_{0}=\frac{\beta T}{k}.$$ (6)

Or it can be rewritten as

$$p_{0}=\frac{\beta }{k\rho C_{V}}\eta_{th}A_{e},$$ (7)

where $A_{e}$ is the specific optical absorption, and $\eta_{th}$ is the percentage that is converted into heat. Here, a Grueneisen parameter (dimensionless) is defined as

$$\Gamma =\frac{\beta }{k\rho C_{V}}=\frac{\beta v_{s}^{2}}{C_{P}}.$$ (8)

Then, Eq. (7) becomes

$$p_{0}=\Gamma \eta_{th}A_{e}.$$ (9)

Or

$$p_{0}=\Gamma \eta_{th}\mu_{a}F,$$ (10)

where $\mu_{a}$ is the optical absorption coefficient and F is the optical fluence.

According to Eq. (10), materials with superior light absorption coefficient $\mu_{a}$ (e.g., graphite $4\;\mu m^{-1}$,^36^^,^^44^^,^^51^ carbon nanotubes (CNT) $70\;\mu m^{-1}$,^37^^,^^38^^,^^52^ gold nanoparticles $80\;\mu m^{-1}$,^53^^,^^54^ and polymer nanoparticles^39^) and large thermal expansion coefficient β [e.g., polydimethylsiloxane (PDMS) 960  μm/m·°C,^37^ epoxy 138  μm/m·°C^36^^,^^44^] could be utilized to boost the absorption and expansion, respectively, subsequently producing acoustic waves with a high pressure.^45^

## 3 Fiber-Based Optoacoustic Neurostimulation

**Fig. 2:**
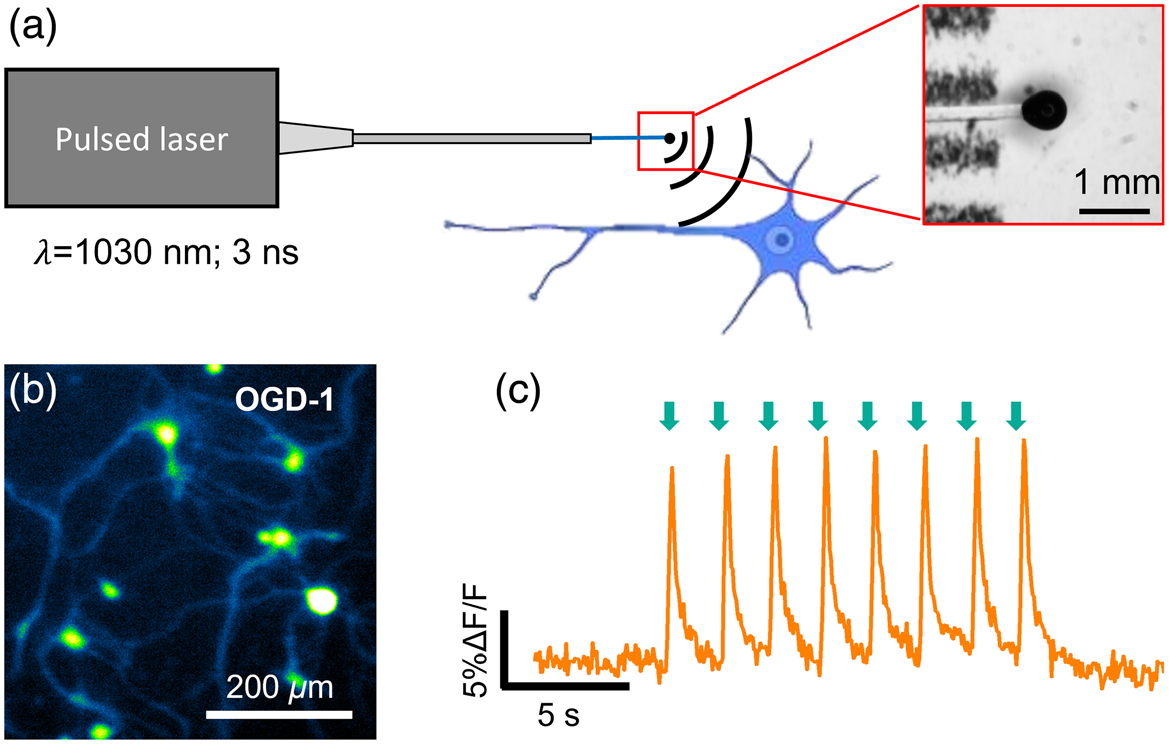
Fiber-based optoacoustic neural stimulation with submillimeter resolution. (a) The concept of optoacoustic neuromodulation through a FOE. Inset is the enlarged FOE tip under stereoscope. (b) Optoacoustic wave induces calcium transients in cultured primary neurons loaded with OGD-1. (c) Calcium trace of a neuron undergone repeated FOE stimulation. Green arrow: stimulation onset. (Adapted with permission from Ref. 36.)

FOEs can serve as miniaturized ultrasound point sources. They are often fabricated by attaching a thin absorption layer on the fiber distal end to convert the pulsed light into acoustic waves via the optoacoustic effect. The highly miniaturized FOEs have been used for drug delivery into cell membrane,^45^ ultrasound imaging,^55^ or integrated into medical devices such as catheters and needles to provide real-time surgical guidance.^44^^,^^56^ In the work of Jiang et al., optoacoustic effect was first exploited for direct neuron stimulation with submillimeter resolution *in vitro* and *in vivo*^36^ (Fig. 2). The FOE-generated acoustic field propagates omnidirectionally away from the optoacoustic coating, resulting a localization of the acoustic field. Specifically, Jiang et al. showed that the FOE with a total diameter of 600  μm generates an acoustic wave, of which the acoustic intensity is attenuated by 61% at 1.0 mm away from the tip underwater. One unique feature of FOE in this work is the two-layer coated fiber tip with ZnO/epoxy as a light diffusion layer and graphite/epoxy as the optoacoustic conversion layer to produce omnidirectional ultrasound. Upon the illumination of a 3-ns 1030-nm pulsed laser, the FOE generated ultrasound with a pressure of 0.48 MPa from the fiber-coated tip. Such localized optoacoustic pressure achieved neurostimulation with ∼500  μm spatial precision in rat primary cortical neurons, confirmed by calcium imaging. All neurons showed activation evident by ~10% calcium fluorescence increase. Successful neural stimulation *in vivo* was demonstrated using local field potential (LFP) recordings in mouse cortex when the FOE was placed on the cortex surface. Strong LFP signals above 40  μV were obtained when the recording electrode was placed <500  μm from the FOE. Importantly, the FOE stimulation at primary somatosensory cortex only induced localized LFP responses at the stimulation site with no response in the contralateral auditory cortex, which showed direct neural activation without the involvement of auditory pathway. Modulation of motor response was achieved by direct stimulation of the motor cortex. The high spatial precision of FOE stimulation also allowed the production of mouse forelimb muscle representation map in the motor cortex.

**Fig. 3:**
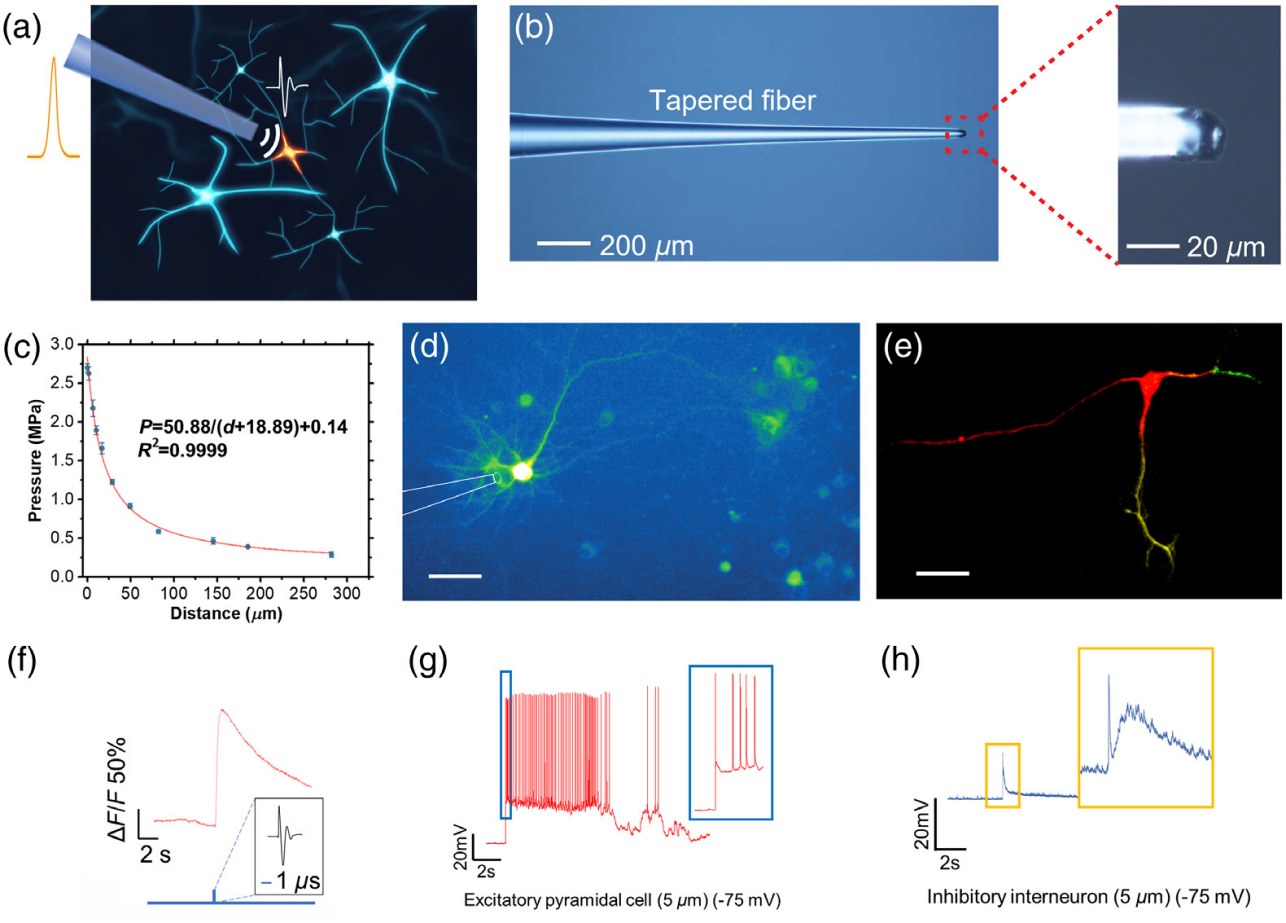
Single cell and subcellular neuron stimulation using TFOE. (a) Schematic of TFOE enabling single neuron stimulation. Nanosecond pulsed laser is introduced into the TFOE to generate acoustic signal via optoacoustic. (b) TFOE consisted of a CNT/PDMS mixture as optoacoustic conversion material. (Left) Optical image of TFOE and (right) zoom-in showing the coated tip. (c) Pressure of the acoustic wave generated as a function of the distance from the TFOE tip. (d) TFOE-induced stimulation of GCaMP6f expressing single neuron. Scale bar: 50  μm. (e) TFOE selectively stimulation of axon (red) and dendrites (yellow and green) of a multipolar neuron. Scale bar: 50  μm. (f) GCaMP6f trace of single neuron stimulated by single pulse from TFOE. (g)–(h), TFOE single neuron stimulation integrated with whole cell patch clamp. Membrane voltage responses in (g) excitatory pyramidal cell and (h) inhibitory interneuron upon TFOE stimulation at a distance of 5  μm are presented. (Adapted with permission from Ref. 37.)

Built upon the work of Jiang et al.,^36^ Shi et al. report a further miniaturized device termed TFOE, capable of targeting single neurons, which is an unprecedented high spatial precision for ultrasound stimulation [Figs. 3(a) and 3(b)].^37^ TFOE was fabricated with an optoacoustic coating of CNT and polydimethylsiloxane (PDMS) mixture on a tapered optical fiber tip and with a total diameter of 20  μm. The produced acoustic pressure shows an attenuation to 1/e at a characteristic distance of 39.6  μm [Fig. 3(c)] with a pressure of 2.7 MPa, allowing single cell stimulation [Fig. 3(d)] and subcellular stimulation of axons and dendrites [Fig. 3(e)]. With the superior temporal controllability of the pulsed laser, a single optoacoustic pulse with a submicrosecond width induced by a single 3-ns laser pulse was capable of single neuron stimulation [Fig. 3(f)]. This single pulse stimulation is much shorter than reported piezo-based ultrasound neuromodulation^57^ which often requires tens of millisecond of acoustic wave.

In addition, TFOE generates a near-field acoustic wave offering minimal mechanical disruption and assuring a stable patching condition. The electrophysiology recording has been simultaneously achieved during TFOE single neuron stimulation using whole cell patch clamp performed in brain slices.^37^ Excitatory pyramidal neurons and inhibitory interneurons were individually targeted. It was found that excitatory pyramidal neurons exhibited lower action potential thresholds, compared with inhibitor interneurons [Figs. 3(g) and 3(h)]. This result revealed cell-type-specific responses to optoacoustic stimulation, which could be attributed to the ion channel distribution among varied cell types. TFOE, as a genetic-free, single-cell stimulation technique, serves as a new tool to understand the mechanism of neuron stimulation.

## 4 Optoacoustic Films: A Flexible and Biocompatible Interface with Nerve System

Biocompatible films have been shown as an essential platform for bioelectronics^58^^,^^59^ and bases for tissue scaffold.^60^ By integrating optoacoustic agents in the biocompatible materials, flexible, and biocompatible optoacoustic films can be designed and developed. For example, optoacoustic films made of single-walled graphene nanoribbons and polyurethane have been shown to enhance the osteogenic differentiation, calcium content, and other regeneration effects in bone engineering.^61^^–^^63^ Such design can also serve as a new neural interface offering multiple functions, including optoacoustic stimulation, structural support, and growth guidance.^64^

**Fig. 4:**
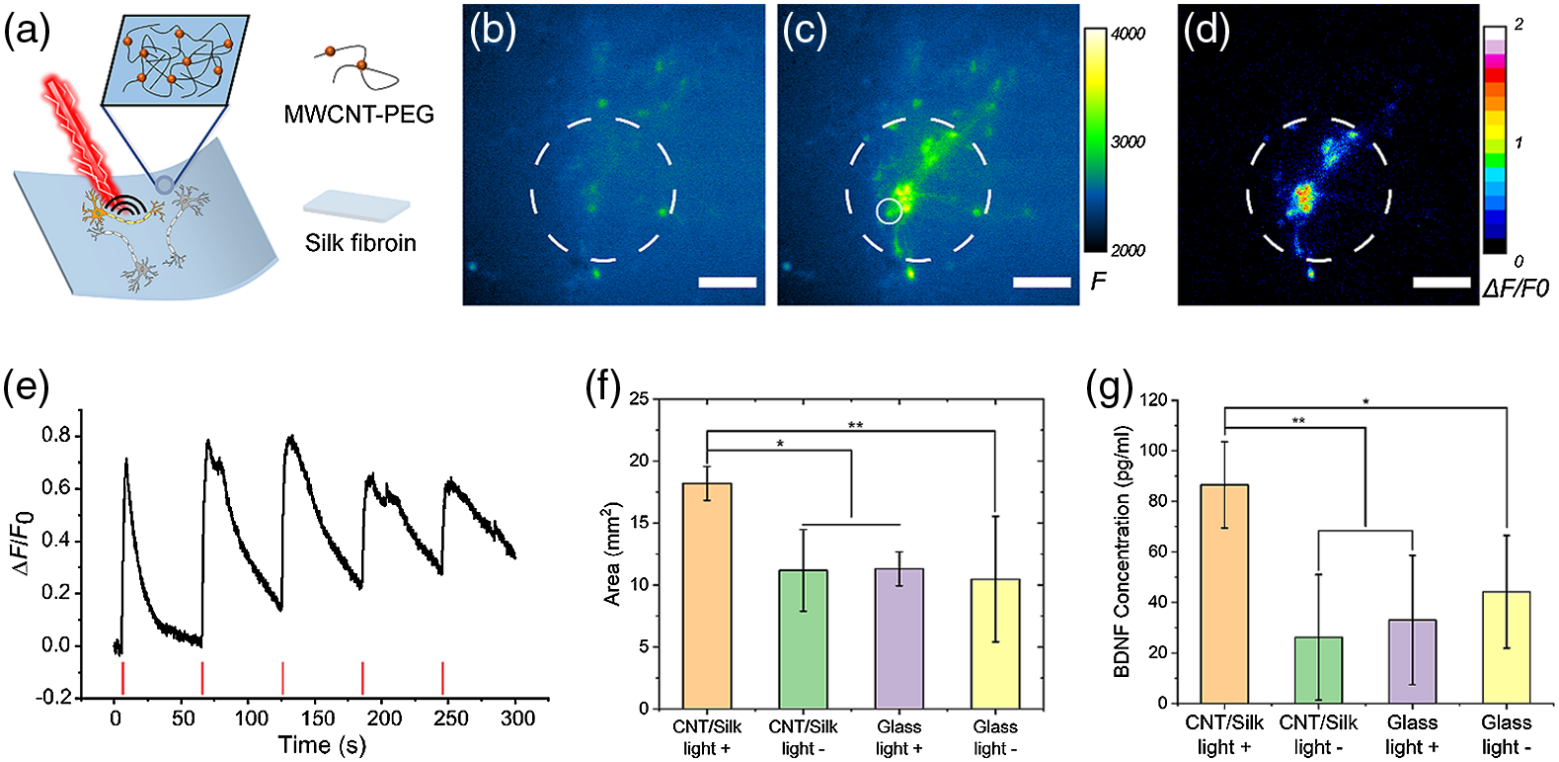
Flexible optoacoustic film for neural stimulation and regeneration. (a) Schematic of optoacoustic CNTs embedded silk film. (b), (c) Calcium image of GCaMP6f transfected rat cortical neurons (b) before and (c) after optoacoustic neural stimulation. (d) Neurons were selectively stimulated defined by the light illumination. Scale bars: 100  μm. (e) Calcium trace of the neuron after undergoing repeated PA stimulation. (f) Average neurite coverage area for DRGs in four groups. CNT/silk film with laser illumination (CNT/silk light +) and without laser illumination (CNT/silk light −). DRGs cultured on a glass bottom dish with laser illumination (glass light +) and without laser illumination (glass light −). (g) Average concentrations of BDNF of PA-stimulated and unstimulated DRGs. (Adapted with permission from Ref. 38.)

Zheng et al. recently described a silk-based optoacoustic film with CNTs using a hydrogel nanocomposite approach^38^ [Fig. 4(a)]. Hydrogels are used as the matrix, providing a biocompatible interface and structural support. Photoacoustic agents, such as CNTs and carbon black, provide light absorption and highly efficient optoacoustic conversion. In this work, the silk fibroin was chosen as it was an FDA-approved biocompatible material and had been shown as a neural scaffold supporting neural adhesion and outgrowth. CNTs have high optoacoustic-conversion efficiency and strong absorption in the second near-infrared (NIR-II, 1000 to 1700 nm) window, which gives it potential in terms of tissue penetration for future *in vivo* applications. Upon excitation of a nanosecond pulsed laser, silk/CNT films were shown to generate optoacoustic waves with a pressure of 0.19 MPa and successfully induced neural activation for the primary cortical neurons and DRG tissues cultured on the film [Figs. 4(b)–4(d)]. Reliable and repeatable calcium activation of GCaMP transfected neurons was confirmed without lasting damage observed [Fig. 4(e)].

Significantly, the optoacoustic film was proven to be capable of promoting neural regeneration through increasing the secretion of brain-derived neurotrophic factors (BDNF) [Figs. 4(f)–4(g)]. Similar promotion effects have been observed using other neural stimulation techniques, for example, electrical stimulation^65^^,^^66^ and optogenetics.^67^ As a light mediated technique, the optoacoustic scaffold eliminates the requirements of wire connections and genetic modifications. Biocompatible and flexible optoacoustic film serves as a new implant for optoacoustic neural stimulation, which is complimentary with FOE devices. Compared with other forms of optoacoustic emitters, the optoacoustic film has several additional features, which could lead to a better performance in specific applications. First, the film optoacoustic materials expand the function volume of optoacoustic neural stimulation. Optoacoustic waves can be generated from the multiple selective areas of the film according to illuminations of the incident laser, rather than being fixed at the tip of a fiber. Second, because of the soft matrix material, flexible films can form conformal attachment with curved brain surface,^68^ ocular tissues,^69^ and peripheral nerves.^70^ Lastly, the film and the 3D structure developed based on the films can support neural adhesion and growth while providing the stimulation function, which could be crucial to tissue engineering applications.

## 5 Neuromodulation Mediated by Optoacoustic Nanotransducers

**Fig. 5:**
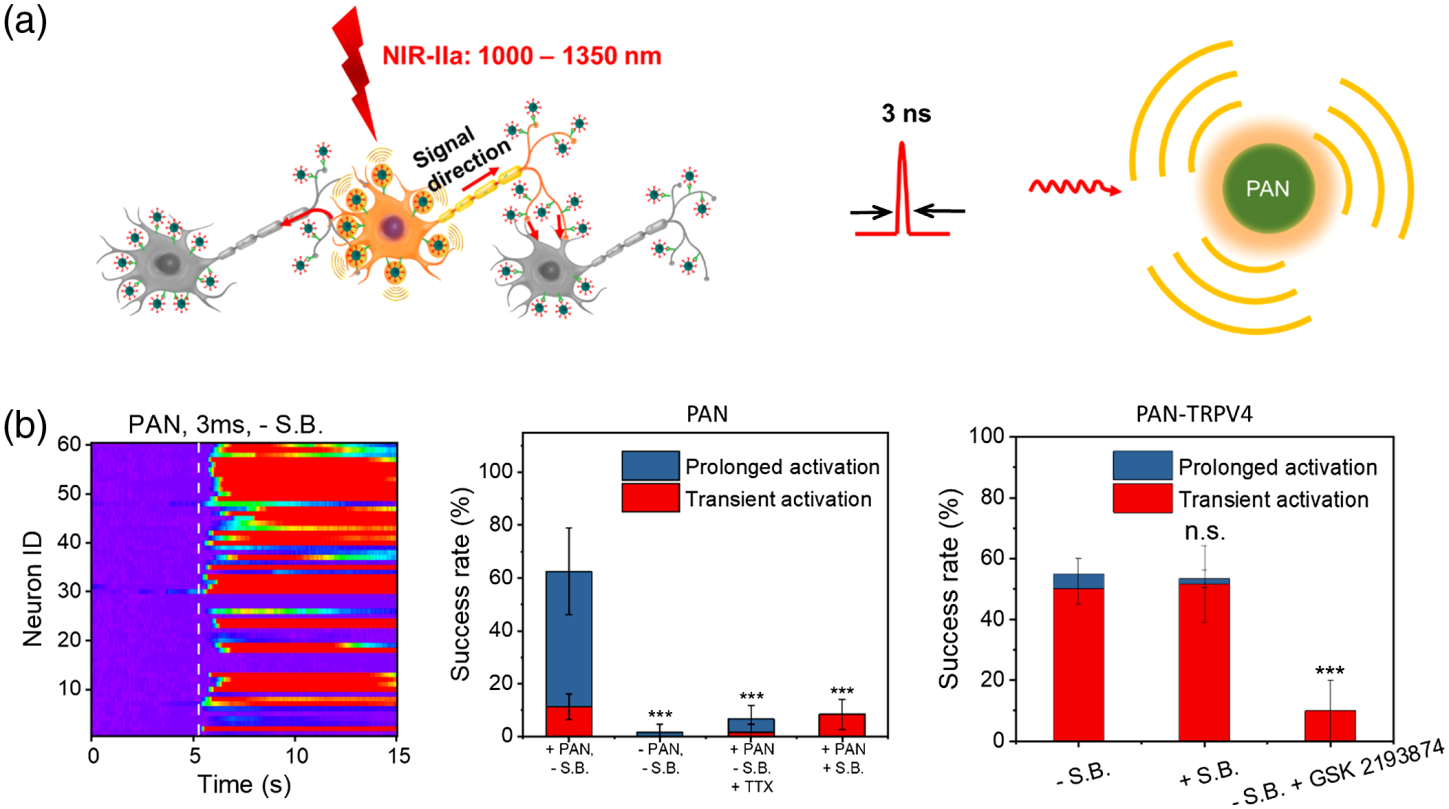
Nanotransducer-mediated optoacoustic neural stimulation. (a) Scheme of the PAN-induced neural stimulation (left) and the PAN generating optoacoustic signal upon nanosecond laser excitation (right). (b) Left: Colormaps of fluorescence changes of neurons stimulated by PANs using the 1030-nm nanosecond laser with a 3-ms pulse train without synaptic blockers. White dash line: laser onset. Middle: Success rate analysis of PAN-induced neuron stimulation profiles with the presence/absence of synaptic blockers or TTX. Right: Success rate analysis of bioconjugated PAN-TRPV4-induced neuron stimulation profiles with the presence/absence of synaptic blocker or TRPV4 channel blocker GSK 2193874. (Adapted with permission from Ref. 39.)

In the past decade, nanoparticle-assisted neuromodulation has seen rapid development. Nanoparticles have been developed to enhance the efficiency of optical,^71^^–^^76^ magnetic,^77^^–^^79^ and acoustic neuromodulation.^80^^–^^82^ Among them, semiconducting polymer nanoparticles represent a new class of nanotransducers due to their unique absorption in the near-infrared (NIR) wavelength, excellent biocompatibility, and programmable biodegradability.^73^ Only recently, optoacoustic neuromodulation using nanoparticles has been achieved^39^ (Fig. 5). In this study, optoacoustic nanotransducers (PANs) were prepared based on semiconducting bis-isoindigo-based polymer (BTII)^73^ and followed by modification with poly(styrene)-b-poly(acrylic acid) (PS-b-PAA) to form water-soluble nanoparticles with the size of ∼50  nm through the nanoprecipitation method. The PANs solution with a concentration of 1  mg/ml generates a robust optoacoustic signal with a peak pressure of 1.36 kPa upon nanosecond laser excitation at 1030 nm. PANs bind to neuronal membrane through a nonspecific charge–charge interaction at ∼43 PANs per soma after coculturing with primary cortical neurons for as little as 15 min, validated by transient absorption imaging. Successful neuronal activation was demonstrated using calcium imaging of GCaMP expressing rat cortical neurons. Millisecond temporal resolution and single cell spatial resolution have been achieved. Success rate was found to be 8.3±5.8% with the presence of synaptic blockers. Importantly, bioconjugation of PANs with the mechanosensitive ion channel TRPV4 antibody formed PAN-TRPV4 and allowed specific targeting of TRVP4 channels, which are abundantly mechanosensitive ion channels expressed in neuron membrane. Stimulation through PAN-TRPV4 further improved the specificity of neural activation, achieved a success rate of 53.3% with synaptic blockers, and significantly altered the response dynamics. *In vivo* stimulation was demonstrated through direct injection of PANs to the mouse motor cortex and laser excitation by an optical fiber. Direct LFP responses were observed at the injection site and motor responses were validated by EMG recordings on the limbs.

The PANs represent a new concept of nongenetic nanoparticle-assisted neuromodulation techniques that have the potential for deep tissue penetration, benefiting from NIR-II excitation. In addition, the antibody targeting strategy also provides opportunities for cell type-specific targeting. Integrated with noninvasive delivery methods, such as ultrasound-induced BBB disruption,^83^^–^^85^ as well as advanced optics for deep tissue light delivery with a tight focus, such as Bessel beam,^86^^,^^87^ PANs open new opportunities for genetic-free and noninvasive neuromodulation with high spatial temporal resolution.

## 6 Mechanisms of Optoacoustic Neuromodulation

**Fig. 6:**
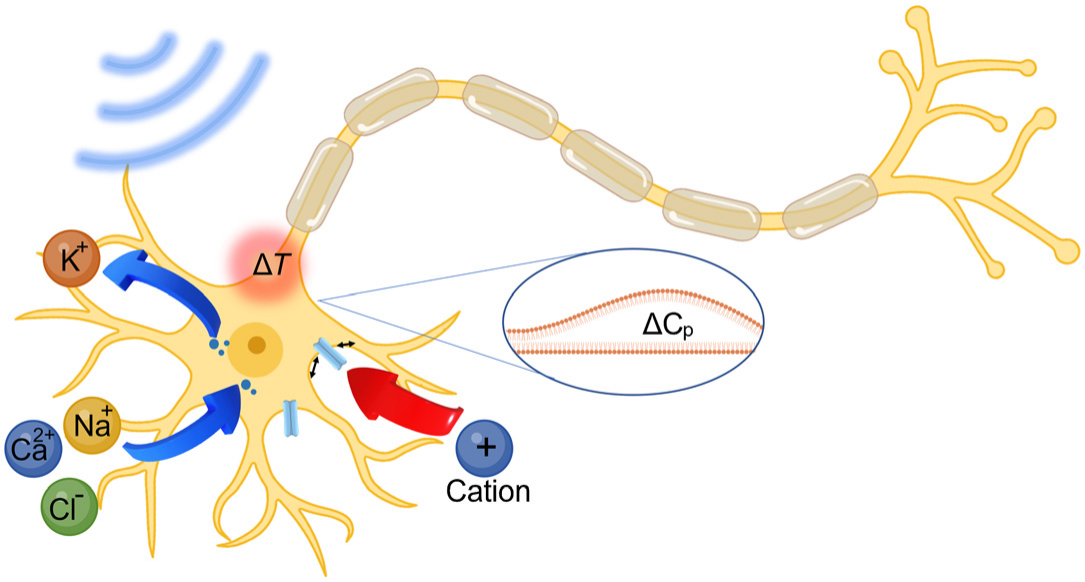
Schematic of possible optoacoustic neuromodulation mechanisms. Blue arrows: sonoporation facilitated ion exchange driven by concentration gradients. Blue dots: transient pores on cell membrane. Red arrow: activation of ion channels induced cellular influx of cations. Inset in the blue ellipse: mechano-electric effect induced altering in membrane capacitance ($\Delta C_{p}$). Red circle: thermal effect induced transient heating on cell membrane. (Created with Ref. 40.)

The biophysical and molecular mechanism of optoacoustic neuromodulation remains largely unknown. Since optoacoustic devices generate acoustic waves in the ultrasonic range, it is likely that the optoacoustic neurostimulation shares the same mechanisms as ultrasound neurostimulation. To date, several hypotheses have been proposed for ultrasound stimulation, including local temperature increase,^88^ transient sonoporation,^45^^,^^89^ intramembrane cavitation,^90^^,^^91^ activation of the auditor pathway,^92^^,^^93^ and activation of the mechanosensitive ion channels^94^^–^^96^ (Fig. 6).

Ultrasonic heating was considered as the primary mechanism for high intensity focused ultrasound modulation.^88^ However, more recent studies using low intensity ultrasound have shown a minimal temperature increase of less than 0.1°C,^28^^,^^97^^,^^98^ much less than the thermal threshold for activation. In optoacoustic neuromodulation, the pressures and frequencies used are within the range of conditions used by ultrasound neuromodulation; however, optoacoustic pulses are delivered with <0.1% duty cycle. Single pulses have been demonstrated for successful neural activation,^37^ in which the heat accumulation effect is minimal. It is also worth noting that the optoacoustic process does involve transient temperature increase, which will be further elucidated in Sec. 7.

Sonoporation involves transient and reversible disruption of membrane integrity due to the mechanical force exerted by the acoustic wave and allows ion exchange across the neuronal membrane and depolarizes the membrane potential. Sonoporation has been demonstrated in fiber and thin film-based optoacoustic devices for applications including drug delivery,^45^ gene transfection,^46^ but its contribution in optoacoustic neuromodulation remains unknown. Future investigations of model membrane system, combining optoacoustic stimulation and whole cell electrophysiology could shed light to this hypothesis.

The involvement of auditory pathway has recently attracted a lot of attention in transcranial ultrasound neuromodulation.^92^^,^^93^ We argue that auditory activation is unlikely in the case of optoacoustic neuromodulation, due to its spatial confinement and extremely low duty cycle. Neural activation demonstrated in cultured primary neurons and *in vivo* by Jiang et al. had provided direct evidence clearly ruling out the auditory involvement in the optoacoustic stimulation.^36^

Lastly, activation of mechanosensitive ion channels has been the most studied hypothesis for acoustic neuromodulation. In an oocyte membrane system, Kubanek et al. recorded ion channel current from mechanosensitive ion channels including TREK-1, TREK-2, and TRAAK and ${\mathrm{Na}}_{v}1.5$.^94^ A few years later, Kubanek et al. identified MEC-4, an ion channel for a touch sensation, as required for ultrasound modulated response in *Caenorhabditis elegans*.^95^ In addition, overexpression of TRP-4, a TRPN family channel, has been shown to enhance ultrasound modulation in *Caenorhabditis elegans* as well.^99^ Using calcium imaging, Gaub et al. investigated the neuronal response to pure mechanical stimuli using atomic force microscope cantilever and identified force and pressure required for transient and sustained activation.^100^ The contribution of various mechanosensitive ion channels was studied using pharmacological manipulation. Using calcium imaging, Yoo et al. looked at activation of various mechanosensitive ion channels by ultrasound stimulation and identified the key contribution of three ion channels including TRPP2, TRPC1, and Piezo1.^96^ Calcium amplification by TRPM4 and voltage gated calcium channels was proposed to be the downstream molecular pathway. However, the incompatibility of ultrasound stimulation with whole cell recording in mammalian neurons hinders further implementing the electrophysiological studies at the single neuron level. The TFOE poses the unique of advantage of compatibility with patch clamp, making it a great tool to overcome this limitation. For future studies investigating the ion channel involvement, TFOE stimulation could be applied with pharmacologically blocking or genetically overexpressing/knocking out specific ion channels, thus potentially unveiling the detailed ion channel dynamics at millisecond time scale.

While they share many similarities, optoacoustic neuromodulation and ultrasound neuromodulation do have some key differences. For ultrasound neuromodulation, continuous wave sonication has been shown to stimulate brain activity in some studies,^101^^,^^102^ and the pulsed delivery paradigm with tone burst consisting of tens of or hundreds of acoustic wave cycles^28^ is more favorable due to a lower risk of tissue heating and lower thresholds for neural activation.^101^ Ultrashort ultrasound pulses with pulse widths up to a few ten microseconds have been reported. Specifically, Kim et al.^103^ applied a 2.1-μs ultrasound pulse at a repetition rate of 1.16 kHz with total duration of ∼63  s to evoke spiking. Weinreb et al.^104^ used a single ultrasound burst of 4  μs for neuron stimulation *in vitro*. Tyler et al.^57^ used ultrasound burst with total duration of 22.7  μs to evoke single action potential *ex vivo*. In comparison, optoacoustic stimulation can be successful under a single or multiple acoustic pulses and each pulse has ∼1  μs duration. Optoacoustic wave often has a broad bandwidth, ranging from 1 to 20 MHz, compared to single frequency acoustic wave from a piezo-based ultrasonic transducer of millisecond time scale, often at sub-MHz frequency for high transcranial efficiency. These differences could lead to important changes in ion channel activation dynamics, threshold, frequency-dependent response, and cell type specificity (if any).

## 7 Optoacoustic Versus Photothermal Effect

The optoacoustic effect is known to be associated with photothermal effect. To investigate how much the temperature increase contributes to the neurostimulation discussed above, temperature increase associated with the successful stimulation conditions for each optoacoustic platform has been studied. For the FOE platform,^36^ the temperature was measured by a miniaturized ultrafast thermal sensor (minimum sensitivity: 0.02°C) directly in contact with the FOE tip surface. The temperature increase was found to be 1.6°C, 0.9°C, 0.5°C for 200, 100, 50 ms laser stimulations, respectively. For the TFOE platform,^37^ temperature on the fiber tip was measured by the same thermal sensor directly in contact with the TFOE tip surface. The temperature increase on the TFOE tip surface was found to be less than 0.02°C under the condition for successful neurostimulation, i.e., a laser pulse train of 1 ms, a laser power at 11.4 mW, and a repetition rate of 1.7 kHz. For the CNT/silk film platform, a laser train duration of 5 ms with a 1.7-kHz repetition rate and pulse energy of 14.7  μJ for neurostimulation only resulted in a temperature increase of 0.15°C. Temperature increase measured directly on the surface of the acoustic sources is all below the previously reported threshold for thermal induced neural activation (ΔT>5°C).^105^ Moreover, for the fiber and FOE, the actual temperature increase at the cell membrane which is around 5 to 100  μm away *in vitro* or further *in vivo* from the emitters is expected to be even lower considering the thermal decay over this distance. These results suggested the optoacoustic effect dominated during the neural stimulation.

For the PANs, through simulation, temperature increase associated with the successful stimulation condition was found to rise to a peak value of 8.4°C on the PANs surface and to 5.0°C at 10 nm away from the PANs surface, respectively. In both cases, temperature decays to the baseline within 10 ns from the peak value. In addition, a control experiment using continuous-wave (CW) laser was performed with PAN systems.^39^ Based on the optoacoustic working principle, the CW laser illumination on nanoparticles is known to induce photothermal effect only, since the thermal/stress confinement criterion is unmet.^106^ We compared the excitation of PANs using nanosecond laser and CW laser at the same power (laser power of $70\;W/{\mathrm{cm}}^{2}$ over 3.9 ms duration for CW). The PANs under nanosecond laser evoked neurostimulation while the PANs under CW laser failed. Moreover, mediated by PANs under CW laser, activation of neurons was only observed when the laser power increased to $397\;W/{\mathrm{cm}}^{2}$ with a duration of 2.5 s. These results confirmed that at comparable power and duration to nanosecond laser conditions, the photothermal effect of PANs was not sufficient to stimulate neurons. The optoacoustic effect played the predominant role for the PANs neural activation.

Notably, multiple studies have reported that light absorption of gold nanoparticles could induce thermal transients that excite neurons.^107^^–^^111^ It is worth noting that the laser conditions used in these studies result in much higher temperature rise and different temperature profiles in the Au nanoparticles compared to what is found for PANs. For example, femtosecond pulsed laser with a repetition rate of 80 MHz at 4 mW applied in de Boer et al. results in a peak temperature increase of 20°C,^107^ significantly larger than the PAN case. In Lavoie-Cardinal et al.’s work, comparable femtosecond pulsed laser conditions with a 80-MHz repetition rate was utilized on gold nanoparticles^108^ with a higher laser pulse energy of $8.75\;\mathrm{mJ}/{\mathrm{cm}}^{2}$ per pulse compared to $10\;\mu J/{\mathrm{cm}}^{2}$ per pulse in de Boer et al.’s work. In other photothermal studies, where CW laser was applied as continuous burst over 1  μs up to 1 ms^24^^,^^111^^–^^114^ and generated heat without optoacoustic effect, the temperature increase on the Au nanoparticle surface raised to a plateau within the first 200 ns in these CW laser cases, with plateaued values at 65.6°C and 10.3°C, respectively.^110^^,^^111^

**Fig. 7:**
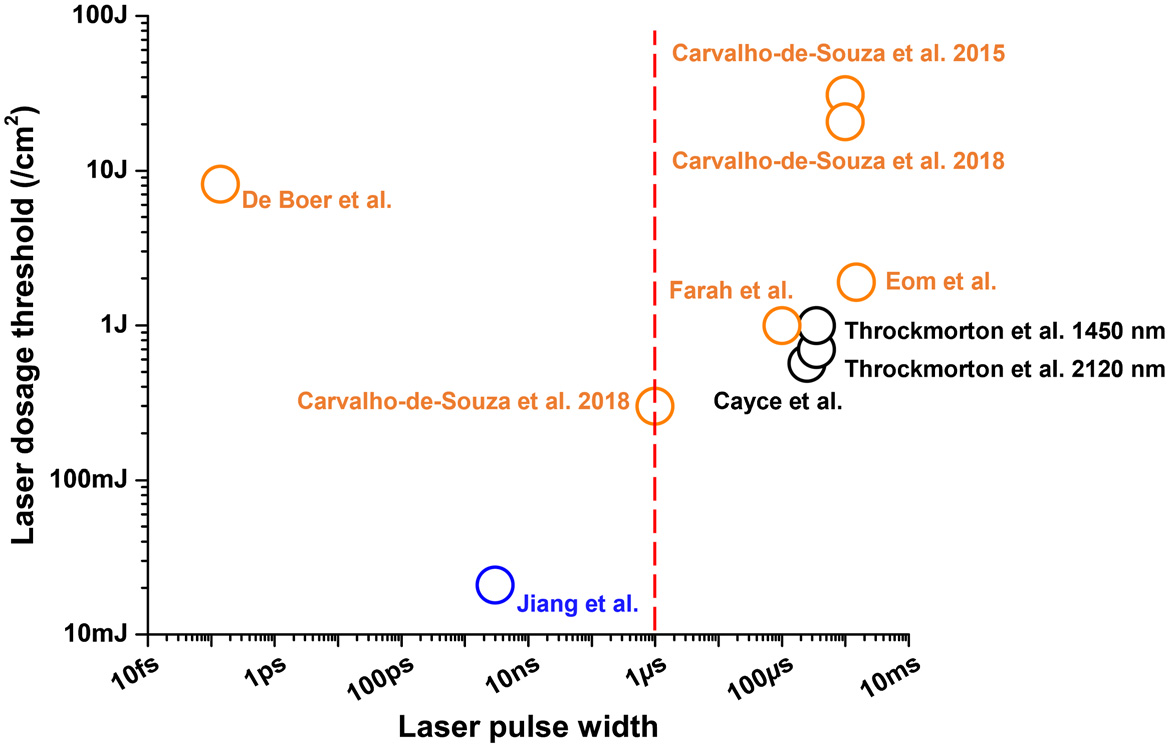
Nanoparticle-based photothermal versus optoacoustic neurostimulation regarding the laser dosage threshold and laser pulse width. Orange circles: nanoparticle-based photothermal using pulsed or CW laser.^107^^,^^111^^–^^114^ Black circle: water-based photothermal neurostimulation with infrared laser.^24^^,^^115^ Blue circle: PANs-based optoacoustic neurostimulation using nanosecond pulsed laser.^39^ Red dash line: suggested 1 *μ*s as the laser pulse width threshold for efficient optoacoustic generation.^116^

To quantitatively compare the different successful stimulation conditions, we summarized the pulsed/CW laser used in nanoparticle-mediated photothermal studies and the pulsed laser used in the PAN optoacoustic study. We also include typical conditions used by infrared neural stimulation (INS), in which water is the absorbing agent.^24^^,^^115^ The overall laser dosage needed for neuron stimulation was plotted corresponding to different laser pulse widths used. As shown in Fig. 7, a vertical red dash line indicates the pulse width of 1  μs, which has been recommended as the laser pulse width threshold to meet the thermal confinement requirement and therefore lead to efficient optoacoustic generation.^116^ A pulse wider than 1  μs generates optoacoustic signals less efficiently compared to shorter pulses. Here, orange circles denote the reported laser conditions for nanoparticle-based photothermal using pulsed or CW laser^107^^,^^111^^–^^114^ and the black circle denotes the laser condition for INS.^24^ The photothermal dosage typically ranges from 300 to $31000\;\mathrm{mJ}/{\mathrm{cm}}^{2}$. As indicated by the blue circle, the PANs-based optoacoustic neurostimulation used the nanosecond pulsed laser^39^ and a laser dose of $21\;\mathrm{mJ}/{\mathrm{cm}}^{2}$, which was far below the dosages for photothermal stimulation. Thus, this comparison suggests that the optoacoustic stimulation enabled by a ns pulsed laser operates at a different laser energy range, with a dosage 10 times smaller than the photothermal stimulation.

## 8 Outlook

As discussed above, optoacoustic neuromodulation is a new and versatile modality that holds the potential to advance the field of acoustic neuromodulation in both basic science research as well as clinical applications. Table 1 denotes the quantitative comparisons of the results obtained across the reviewed technological variants, including stimulation conditions, such as laser dose, pulse number, and stimulation outcomes, such as temporal and spatial response features. We note when selecting the best platforms for applications, how the PA platform will interface with the neural system is also a consideration. FOC and TFOE offer implants that can target deep in the neural tissue as the delivery of light is enabled by the fiber. The fibers do not need to place in contact with the selected cell. The film design offers a strategy that can be used as a functional 3D bioscaffold.^64^ PAN is a potential noninvasive solution. Overall, optoacoustic neuromodulation poses a number of advantages over its ultrasonic counterpart including higher spatial temporal resolution, minimal thermal accumulation, and broad bandwidth, which make it suitable for region-specific modulation in animal models and even in human patients. On the other hand, optoacoustic neuromodulation is still at an early stage of development, and there are several challenges to be addressed by future studies.

**Table 1:** Optoacoustic neuromodulation platforms with quantitative comparisons across technological variants.

	Spatial precision	Distance to neuron	Success rate	Laser dose ($J/{\mathrm{cm}}^{2}$)	Pulse number	Calcium indicator	Max ΔF/F	Latency
FOC	Sub-mm	<500 μm	≈100% (SB-)	8.3	180	OGD	5%	16 ms (LFP)
TFOE	40 μm	5 to 10 μm	—	5.1	1 to 4	GCaMP6f	20% to 150%	4 ms (PC)
PAN	20 μm	>10 nm	53.3% (SB+)	0.021	10	GCaMP6f	11% to 60%	128 ms (EMG)
CNT/Silk	200 μm	>10 nm	96.1% (SB-)	0.029	5	GCaMP6f	75% to 180%	<50 ms (FL)

Note: SB, synaptic blocker; LFP, local field potential; PC, patch clamp; EMG, electromyography; FL, fluorescence imaging

Toward fundamental studies, the mechanism of optoacoustic neuromodulation needs further investigations. Mechanistic studies of ultrasound stimulation have established plausible hypotheses of acoustic neuromodulation. Yet considering the unique features of optoacoustic emitters, these hypotheses need to be reexamined in the context of optoacoustic stimulation. Taking advantages of the compatibility of TFOE and patch clamp recordings, future studies combining TFOE stimulation with pharmacological and genetic manipulations will provide insight into the cellular and molecular mechanism of optoacoustic stimulation. Also, optoacoustic emitters are versatile and metal free, which allows integration of optoacoustic stimulation inside an MRI scanner to study the effect of acoustic stimulation on the whole brain scale.

Toward clinical applications, since it does not require any genetic modification, optoacoustic neuromodulation is suitable for precise modulation of neural activities in human patients. To further adapt the optoacoustic stimulation to clinical application, such as DBS or retina stimulation, further electrophysiology investigation is needed. Specifically, shorter latency and higher frequency of stimulation often desired for the treatment are to be demonstrated. Meanwhile, many opportunities are opened up. It is possible to further engineer the ultrasound field generated to produce a focused optoacoustic wave. For example, focused optoacoustic fields can be produced via developing curved optoacoustic film, thus achieving excitation of neurons transcranially. Engineering the emitters to generate desired acoustic fields^117^^–^^119^ to match specific brain nucleus or targets, such as substantia nigra, will allow for customized and spatially confined acoustic DBS without affecting surrounding brain regions. Toward noninvasive optoacoustic deep brain modulation with high spatial resolution, another possible solution is by noninvasive delivery of PANs and light into deep tissue as described previously. Besides, for clinical application, the high precision photoacoustic stimulation can be used as a surgical tool or an implant for precise stimulation of a single nerve. For example, TFOE can be used to assist a SDR surgery, during which the precise stimulation of the individual dorsal root nerves is needed to identify the abnormal one. The dorsal root nerves can be as small as 0.27±0.13  mm.^120^ Due to electrical current spreading, the commonly used electric stimulation lacks sufficient spatial precision desired. Such precision is even more challenging in children with cerebral palsy as their nerves are finer. TFOE provides the superior stimulation precision needed. In addition, multiplexed emitters in the form of fiber or film-based arrays can potentially be used for stimulation. This can be applied to RGC as a potential visual prosthesis. The current retinal prostheses based on a microelectrode array or photovoltaic array suffered from the poor resolution, typically millimeter or hundreds of micrometers due to current spreading.^121^ The photoacoustic stimulation offers a ∼100 or sub 100 micrometer resolution, opening up a potential to achieve high precision retina stimulation as a promising treatment for vision conditions, such as age-related macular degeneration.
